# Advances in the Isolation and Purification of Fungal Mycotoxins: From Classical Extraction to Precision Strategies

**DOI:** 10.3390/molecules31122170

**Published:** 2026-06-20

**Authors:** Larisa E. Botte, Alena N. Alekseeva, Nikita A. Vasilev

**Affiliations:** 1 Advanced Engineering School, ITMO University, St. Petersburg 191002, Russia; 2 All-Russian Institute of Plant Protection, Podbelskogo St., 3, Pushkin, St. Petersburg 196608, Russia

**Keywords:** secondary metabolites, mycotoxins, purification, isolation, cocrystal, cybernetic membrane, supercritical fluid extraction, AI

## Abstract

Mycotoxins are fungal secondary metabolites with dual significance: they threaten health via food contamination yet hold potential as biopesticides. Their isolation from complex matrices remains a critical challenge. This review analyzes classical methods (liquid–liquid extraction, SPE including QuEChERS, chromatography). Traditional techniques suffer from poor selectivity, multi-step processing, large toxic solvent volumes, and matrix effects. As alternatives, emerging strategies based on rational design are considered: directed cocrystallization, supercritical fluid extraction, smart MOF/COF membranes, and AI integrated with physicochemical modeling. The concept of “precision” extraction enabling prediction of target isolation at the molecular level is developed. Recommendations for standardizing experimental reporting to create machine-readable datasets for neural networks are provided. The review concludes that while most still require experimental validation for mycotoxins, these approaches point toward selective, sustainable mycotoxin isolation technologies for analytical control and pure standard production.

## 1. Introduction

**Figure 1:**
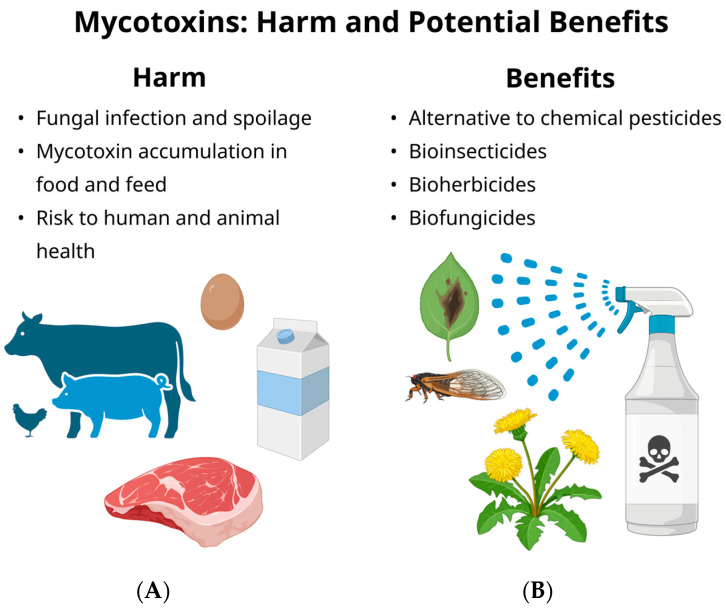
The dual nature of mycotoxins. (**A**) Adverse effects: Fungal infection causes spoilage of agricultural produce and accumulation of mycotoxins in food and feed, posing serious risks to human and animal health. (**B**) Beneficial applications: Mycotoxins and toxigenic fungi can be utilized as sustainable alternatives to conventional pesticides, including bioinsecticides, biofungicides, and bioherbicides.

Mycotoxins are low-molecular-weight secondary metabolites (typically below 1000 Da) produced by toxigenic fungi, in which they serve an ecological role in suppressing competing organisms. Their principal significance for humans, however, stems from food and feed contamination following fungal colonization of crops, which can cause mutagenic, carcinogenic, and genotoxic effects in animals and humans (Figure 1) [1,2,3,4]. To date, more than 400 mycotoxins from approximately 350 fungal species have been described, among roughly 1000 bioactive fungal metabolites [1,2]. The most prevalent and well-studied include aflatoxins (AF), ochratoxin A (OTA), Alternaria toxins, fumonisins (FUM), trichothecenes, citrinin (CIT), zearalenone (ZEN), ergot alkaloids, and patulin (PAT). Collectively, these compounds display a broad spectrum of toxicity—carcinogenic, nephrotoxic, immunotoxic, estrogenic, neurotoxic, and cytotoxic—largely mediated by DNA damage, inhibition of protein synthesis, and oxidative stress [3,4,5,6,7,8].

Because of this toxicity, mycotoxin contamination is a serious problem for the agro-industrial sector and for food safety, the latter aggravated by the carry-over of toxins into animal-derived products. Reliable identification and quantification of mycotoxins is therefore a priority, and analytical methods for this purpose continue to be developed and refined [9,10].

At the same time, mycotoxins are not merely undesirable contaminants. Owing to their potent and frequently unconventional modes of action, several are promising candidates for biopesticides—bioherbicides [11,12,13,14,15,16] and bioinsecticides [17,18,19]—offering more sustainable alternatives to conventional agrochemicals (Figure 1). This dual role makes the recovery of individual mycotoxins in pure form important both for analytical reference standards and for the development of mycotoxin-based agents.

In either case, the isolation and purification of mycotoxins is a critical bottleneck. Mycotoxins typically occur in complex biological matrices, and their recovery is complicated by several interrelated challenges. First, their chemical heterogeneity precludes any single universal protocol for extraction and detection [20]. Second, the fungal matrix is rich in lipids, proteins, polysaccharides, and pigments that compete for sorbent and solvent binding sites, induce pronounced matrix effects in LC–MS/MS (ion suppression or enhancement), and hinder purification [21]. In addition, target mycotoxins are frequently present at trace levels, demanding efficient preconcentration with minimal analyte loss. Third, many mycotoxins are prone to degradation or transformation under harsh conditions (elevated temperature or extreme pH), which reduces recovery and alters the metabolite profile. Consequently, no conventional technique offers, on its own, the combination of selectivity, sensitivity, and breadth required for multi-mycotoxin analysis, and isolation usually relies on laborious multi-step workflows.

These limitations motivate a shift in perspective. Beyond critically reviewing the constraints of conventional extraction and separation techniques, this review advocates a transition from empirical trial-and-error toward what we term “precision” extraction—the rational, molecular-level design of selective isolation. We review emerging strategies aligned with this paradigm, including directed cocrystallization, supercritical-fluid extraction, smart MOF/COF (“cybernetic”) membranes, and the integration of artificial intelligence with physicochemical modeling, and we discuss the experimental-reporting practices needed to make such approaches data-driven and reproducible.

## 2. Extraction of Mycotoxins

**Figure 2:**
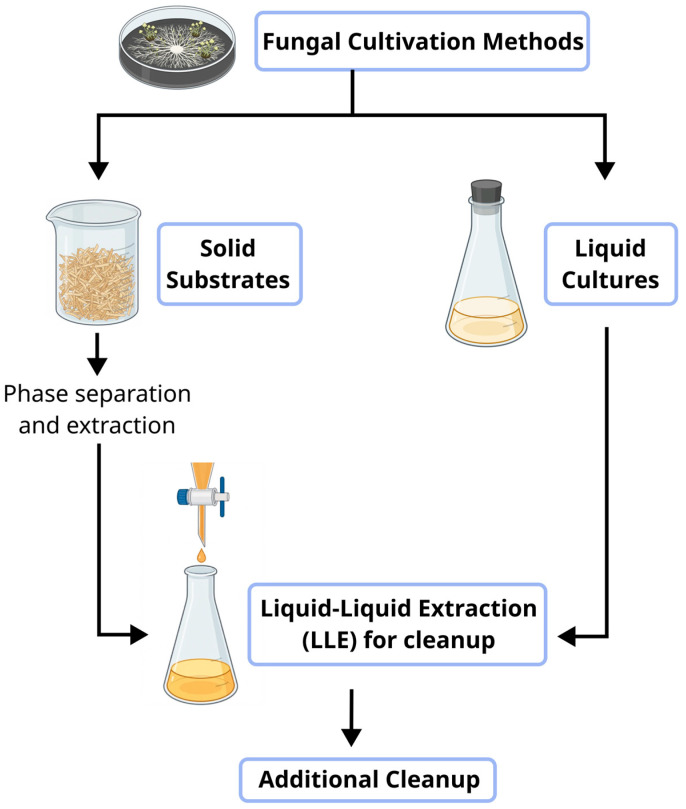
Scheme of sample preparation for mycotoxin analysis from different fungal cultivation methods.

Microscopic fungi can be cultivated on both solid substrates and in liquid nutrient media. The sample preparation strategy for mycotoxin analysis depends on the culture medium. For liquid cultures, liquid–liquid extraction (LLE) using immiscible solvents is typically employed to separate mycotoxins from medium components. For solid substrates (e.g., grains, feed, solid media), extraction is performed using polar solvent mixtures, such as ethanol–water or methanol–water. Following the removal of the volatile organic component, the resulting aqueous phase is subjected to LLE for further purification and concentration of mycotoxins (Figure 2).

**Figure 3:**
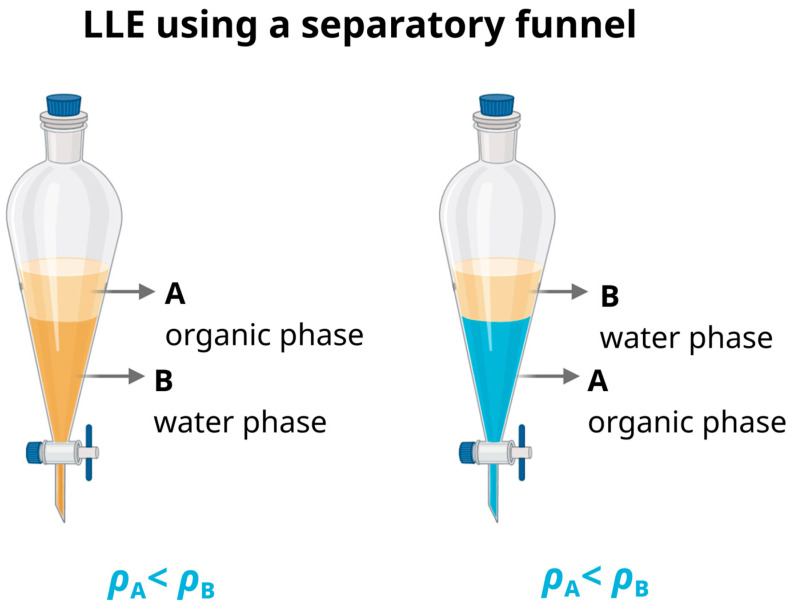
Phase distribution in liquid–liquid extraction. Phase A (extractant) is an organic solvent, immiscible or partially miscible with the first phase. Phase B (original, aqueous) is typically a liquid sample (plant tissue extract, food homogenate, biological fluid, etc.), often aqueous or aqueous-organic. The layering of A and B in a separatory funnel depends on their density (ρ).

Liquid–liquid extraction is based on the redistribution of analytes between two immiscible or partially miscible liquid phases according to Nernst’s distribution law. The partition coefficient K is defined as:

(1) $$K=\frac{{\left[HA\right]}_{A}}{{\left[HA\right]}_{B}}=\frac{C_{A}}{C_{B}}$$

where C is the concentration of a substance dissolved in two immiscible and equilibrated liquid phases A (organic) and B (aqueous) (Figure 3) at a given temperature, K is the partition coefficient, and [HA] is the concentration of the neutral molecule of a weak acid in the aqueous and organic phases. Extraction is readily feasible when K > 1, i.e., when the substance dissolves significantly better in the extracting solvent (C_A_) than in the other (C_B_).

For some mycotoxins that are weak acids [1] and can dissociate in water, the generalized (effective) distribution coefficient D (distribution ratio) is used. D accounts for the fact that the aqueous phase contains not only the neutral form HA, but also the ionized form A^−^, which scarcely transfers into the organic phase (HA ⇌ H^+^ + A^−^). It is expressed as follows:

(2) $$D\;=\;\frac{{\left[HA\right]}_{A}}{{\left[HA\right]}_{B}+{\left[A^{-}\right]}_{B}}$$

The relationship between D, K, and the dissociation constant K_a_ is described by the formula:

(3) $$D=\frac{K}{1\;+\;10^{\left(pH-pK_{a}\right)}}$$

where K is the partition coefficient, and pK_a_ is the acid dissociation constant. It follows that D decreases as pH rises when the acid ionizes. Thus, to increase the extraction of a weak acid, the target mycotoxin—i.e., to convert the weak acid into its HA form—it is necessary to acidify the medium so that pH << pK_a_.

**Figure 4:**
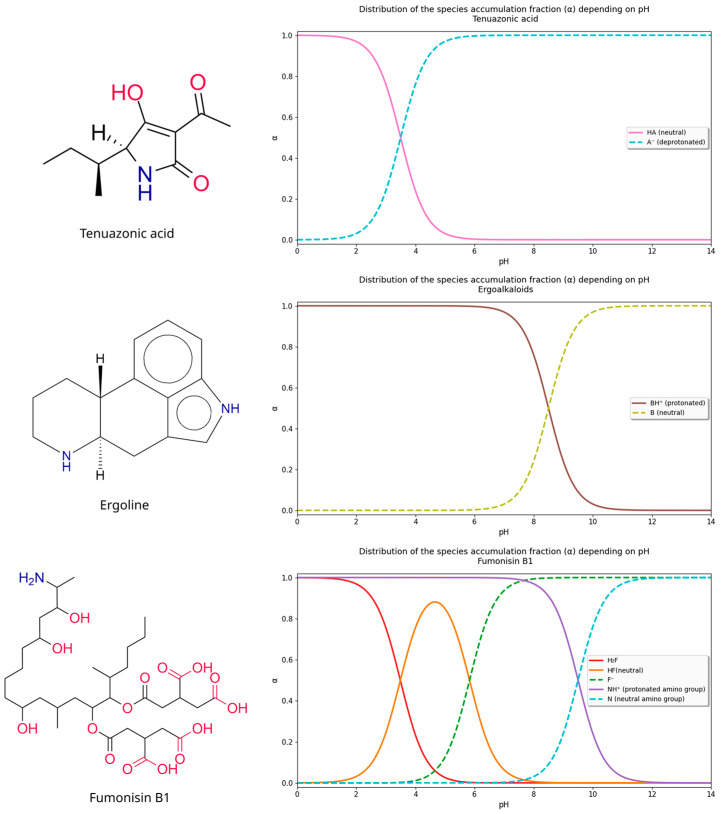
Representative mycotoxins with distinct acid–base properties. Tenuazonic acid (enol form) acts as a weak acid due to the presence of a carboxyl group prone to deprotonation [25]. Ergot alkaloids (e.g., ergoline derivatives) function as weak bases, containing nitrogen atoms in heterocyclic systems capable of protonation; their optimal extraction pH is approximately 8.5 [26]. Fumonisin B1 is an ampholyte possessing both basic amino and acidic carboxyl groups, which allows for zwitterion formation. Enhanced extraction efficiency for free fumonisin is typically observed in buffers at pH 7.5 [27].

There are also mycotoxins that are weak bases and ampholytes. For example, ergot alkaloids contain amine groups and often amide linkages [22], and therefore can be classified as weak bases: they carry a positive charge in an acidic environment and are neutral in an alkaline one [23]. Fumonisins (e.g., Fumonisin B1) are classified as ampholytes because they contain both carboxyl/acyl fragments (acidic centers) and primary/secondary amino groups (basic centers) and are stable in the pH range 4.8–9.0 [24] (Figure 4).

Solvent choice is governed by polarity. By the “like-dissolves-like” principle, non-polar toxins are most soluble in non-polar solvents; however, purely non-polar solvents are immiscible with aqueous matrices and cannot disrupt polar toxin–matrix interactions, and multi-mycotoxin samples span a range of polarities. Solvents of intermediate polarity are therefore usually the most effective compromise [28]. Analyte hydrophobicity is conveniently expressed by the octanol–water partition coefficient (logP); representative mycotoxins and suitable solvents are summarized in Table 1.

### 2.1. Extraction Process

**Figure 5:**
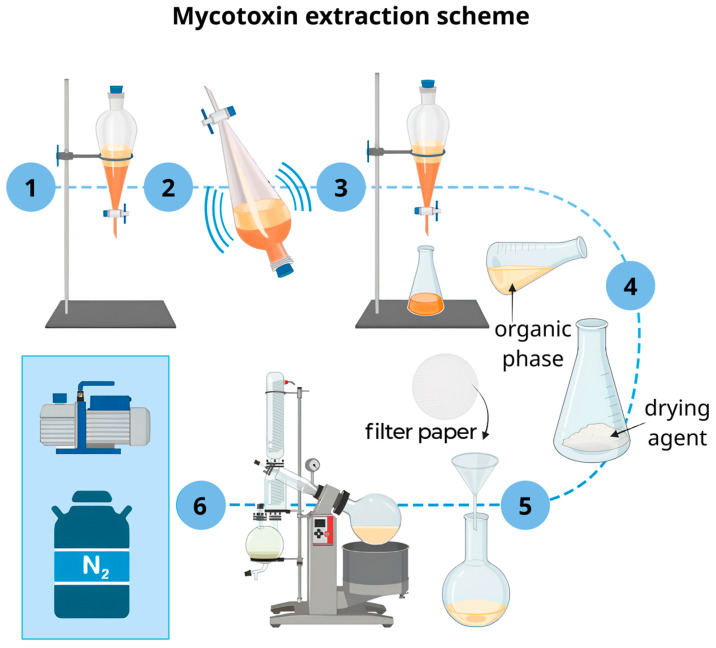
Scheme for mycotoxin extraction. (1) Sample loading into a separatory funnel. The aqueous extract and the selected organic solvent-extractant are placed into a separatory funnel. (2) Intensive shaking and reaching distribution equilibrium. The phase mixture is shaken vigorously with periodic venting; afterward, it is left to stand until complete layer separation. (3) Phase separation. The lower (usually aqueous) phase is drained off, and the organic phase (containing the dissolved mycotoxin) is collected in a clean flask. (4) Drying of the organic extract. The organic phase is transferred to a flask with a desiccant to remove traces of dissolved water. (5) Filtration. The dried extract is filtered through filter paper to remove desiccant particles. (6) Solvent removal and analyte concentration. The filtrate is evaporated using a rotary evaporator under reduced pressure with gentle heating or under a stream of inert gas (nitrogen), yielding a mycotoxin concentrate.

The original liquid (aqueous phase) and the organic solvent are vigorously mixed to ensure the maximum transfer (extraction) of the target toxins from the initial phase into the organic one. The mixture is then allowed to settle in a separatory funnel or is centrifuged. Due to their different densities and immiscibility, the phases separate into two distinct layers. The layer containing the organic solvent and the extracted toxins is carefully decanted or drained (Figure 5).

To obtain the mycotoxin or mycotoxin mixture, the organic solvent must be removed. The organic solvent may contain a small amount of dissolved water. To prevent potential damage to analytical equipment or to ensure the purity of the final concentrate, water is removed from the organic extract using an anhydrous desiccant. The desiccant must not react with the target mycotoxins or the solvent and must be easily removable by filtration. Anhydrous Na_2_SO_4_ and MgSO_4_ are commonly used, as they are applicable for drying most organic compounds and act by forming crystal hydrates [33]. However, MgSO_4_ is sensitive to acids in the solvent, so it is better not to use it when isolating acidic mycotoxins.

Further removal of the organic solvent is based on the relative non-volatility of mycotoxins compared to the more volatile organic solvents (organic solvents have lower boiling points). This is achieved by rotary evaporation, evaporation under a stream of inert gas, or vacuum evaporation. As a result of these processes, a dry residue or a small concentrated volume remains at the bottom of the vessel—this is the extracted mycotoxins or mycotoxins, mixed with a small amount of other lipophilic substances that also entered the extractant. To optimize this process, methods exist that simultaneously extract and separate toxins: for example, solid-phase extraction with activated carbon [34] and graphene oxide [35], ultrasound-assisted extraction, pressurized liquid extraction, magnetic solid-phase extraction [36] and many others.

However, even after all extraction steps, a number of technical challenges remain. Liquid–liquid extraction (LLE) is a labor-intensive, multi-stage process, and at each step there is a risk of partial loss of the target mycotoxin or alteration of its chemical properties. Moreover, this method does not always ensure complete and selective recovery of the analytes.

In practice, the aqueous sample and organic solvent are mixed vigorously to drive analyte transfer and then allowed to separate by settling or centrifugation; the organic layer containing the mycotoxins is collected, dried over an anhydrous desiccant such as Na_2_SO_4_ [33], and concentrated by rotary evaporation or under a stream of inert gas to give a mycotoxin extract (Figure 5). The dry residue contains the target mycotoxins together with co-extracted lipophilic matrix components. To combine extraction with partial cleanup, several integrated variants are used, including solid-phase extraction on activated carbon [34] or graphene oxide [35], ultrasound-assisted and pressurized-liquid extraction, and magnetic solid-phase extraction [36]. Even so, LLE remains labour-intensive and multi-stage, and at each step the target analyte may be partially lost or chemically altered; its principal limitations are summarized below.

### 2.2. Limitations of LLE for the Isolation of Mycotoxins

LLE requires substantial volumes of organic solvents (e.g., acetonitrile, chloroform, methanol), leading to high costs for procurement and disposal. Moreover, many of these solvents pose environmental hazards due to their volatility and toxicity. The extraction procedure is time-consuming and demands considerable laboratory resources. Organic solvents extract not only the target mycotoxins but also numerous other lipophilic compounds from the sample matrix, such as fats, pigments, and carbohydrates [21]. This results in “dirty” extracts, which induce matrix effects during HPLC analysis and compromise the accuracy of mycotoxin quantification. Certain mycotoxins are highly polar and may be incompletely extracted by organic solvents, particularly when suboptimal solvent ratios or acidification/alkalinization modifiers are not employed. Additionally, losses of non-polar target mycotoxins may occur when medium-polarity solvents are used. Mycotoxins may also partially adsorb onto drying agents or remain on the filter. Extraction of samples with high protein, fat, or starch content often leads to the formation of stable emulsions. These emulsions are extremely difficult to break, impeding clear phase separation and resulting in the loss of valuable analytes [37].

Cultivation of fungi typically yields relatively small quantities of mycotoxins, which must be recovered without loss. Often, the extraction process yields a mixture of mycotoxins that differ in polarity (e.g., trichothecenes) and chemical properties (e.g., acids and neutral molecules). Further complexity arises from the presence of structural and stereoisomers, which may exhibit similar solubilities and analogous behavior during conventional liquid–liquid extraction and sorbent-based purification steps. These isomers are frequently co-extracted and persist through identical clean-up stages, leading to cross-contamination of fractions and complicating the isolation of high-purity individual standards. Consequently, there is a pressing need for alternative approaches capable of simultaneously isolating and separating mycotoxins.

## 3. Classical Methods for Separating Mycotoxins

**Figure 6:**
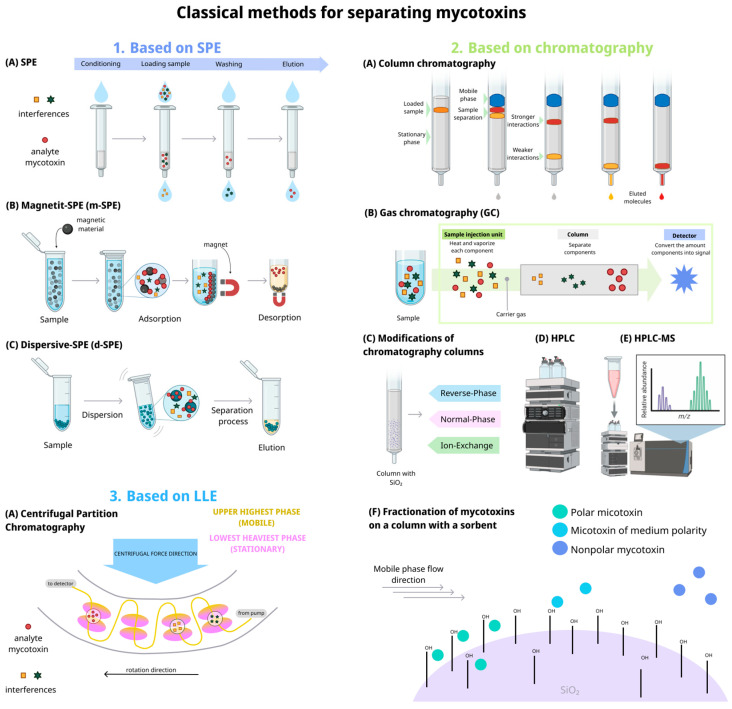
Classical methods for the separation of mycotoxins. Schematic representation of the main methodological groups employed for the isolation and purification of mycotoxins from complex matrices. Methods based on solid-phase extraction (SPE) include conventional SPE (**1A**), magnetic SPE (**1B**), and dispersive SPE (d-SPE) (**1C**). Methods based on chromatographic separation encompass column chromatography (**2A**), gas chromatography (**2B**), various modifications of chromatographic columns (**2C**), high-performance liquid chromatography (HPLC) (**2D**), liquid chromatography–mass spectrometry (HPLC-MS) (**2E**), and preparative fractionation of mycotoxins using sorbents of varying polarity (**2F**). Methods based on liquid–liquid partitioning (LLE) are represented by centrifugal partition chromatography (CPC) (**3A**). All of the presented approaches differ in their separation principles, types of stationary phases, and degrees of selectivity; nevertheless, they remain fundamental to modern strategies for mycotoxin analysis.

The analyte obtained after extraction often contains a mixture of mycotoxins or impurities. The extraction process yields multicomponent mixtures in which the concentration of target toxins is typically low, while background compounds are abundant. The isolation of target mycotoxins necessitates the application of separation methods. Classical techniques for the separation of mycotoxins, along with their descriptions, are presented below (Figure 6).

### 3.1. Methods Based on Solid-Phase Extraction

These methods are based on the interaction of analytes with a solid sorbent phase. The purification or separation of mycotoxins occurs due to differences in the affinity of compounds for the stationary phase and the solvent, arising from variations in polarity, charge, or molecular structure. Such methods are employed during sample preparation to remove interfering substances and concentrate toxins. They are characterized by simplicity, reproducibility, and the ability to concentrate analytes; however, their effectiveness depends on the appropriate selection of sorbent and elution conditions. Their limitations include partial non-specificity of sorbents, matrix effects, and a lack of universal applicability when analyzing mixtures of chemically diverse mycotoxins.

#### 3.1.1. Solid-Phase Extraction (SPE)

Solid-phase extraction (SPE) is considered the “gold standard” for sample preparation in analytical chemistry. Separation occurs due to the differential affinity of substances dissolved in a liquid (mobile phase) for the solid sorbent packed within a small cartridge (stationary phase), through which the sample is passed. As a result, either the target analytes (mycotoxins of interest) or the undesirable matrix components are retained on the stationary phase. If the retained fraction contains the target analytes, they are subsequently eluted from the stationary phase using a suitable eluent. The SPE technique is analogous to column chromatography and is commonly employed as a sample preparation step prior to the analysis of mycotoxins by liquid chromatography–tandem mass spectrometry (LC-MS/MS) [38].

A variety of commercial sorbents are available and are typically categorized into four groups based on the polarity of the adsorbent [39]: (1) adsorbents for non-polar or weakly polar compounds, such as octadecylsilane (C18) or C8 [40]; (2) adsorbents for polar compounds, including CN, NH_2_, Florisil, and amino/phenyl-bonded silica; (3) ion-exchange adsorbents, such as SAX, PRS, PSA, and ion-exchange resins; and (4) additional adsorbents used in commercial columns with specialized materials, such as Oasis Hydrophilic Lipophilic Balance (HLB) and immunoaffinity columns (IAC), which are commonly employed for the isolation of mycotoxin groups from food samples [41].

For multi-class analysis, either column combinations are required—though immunoaffinity columns offer limited reusability (typically 1–3 cycles)—or multi-IAC approaches must be employed, both of which increase cost and procedural complexity. Adsorbents designed to selectively retain only relatively polar or non-polar mycotoxins are inefficient for the simultaneous comprehensive isolation of mycotoxins with diverse chemical properties. Furthermore, mycotoxins are typically present at very low concentrations within complex matrices (e.g., grains, nuts, feed, beverages) rich in lipids, proteins, sugars, and phenols. Even when ion-exchange adsorbents are used, a significant proportion of co-extracted matrix components remain in the extracts, compromising the accuracy of LC–MS/MS analysis and potentially leading to matrix effects.

This has motivated active research into novel, non-conventional types of adsorbents, including molecular recognition-based polymers—such as molecularly imprinted polymers [42] and aptamers [43], nanoparticle-based adsorbents (e.g., graphene and its composites, activated carbon and its composites), bonded silica-based adsorbents (e.g., humic acid-bonded silica [41]), and other materials [44] as well as hypercrosslinked polymers, such as those synthesized from heterocyclic phenylimidazole monomers.

#### 3.1.2. Dispersive Solid-Phase Extraction (d-SPE)

d-SPE is a variant of solid-phase extraction in which the sorbent (or a mixture of sorbents) is dispersed directly into the extract (or following extraction), most commonly after phase separation or another preliminary cleanup step. After dispersion and interaction of the sorbent with the matrix and mycotoxins, the sorbent is separated (by centrifugation, magnetic field, sedimentation, or other means and the purified analytes are recovered). d-SPE is frequently employed as a cleanup stage following QuEChERS; after salt-induced phase separation, d-SPE is used to remove residual matrix interferences [45].

Several modifications of the d-SPE method are commonly employed in mycotoxin analysis, namely dispersive micro-solid-phase extraction (D-μ-SPE) and magnetic solid-phase extraction (m-SPE). D-μ-SPE utilizes small quantities of micro- or nanomaterials and can be further enhanced by ultrasound- or vortex-assisted techniques. m-SPE combines magnetic properties with functional materials—such as magnetized carbon nanoparticles, functionalized magnetic nanoparticles, and magnetized nanoporous materials [44,46]. Challenges associated with these approaches may arise from complex sample matrices, trace-level analyte concentrations, and the diversity of physicochemical properties exhibited by mycotoxins [47].

#### 3.1.3. QuEChERS

**Figure 7:**
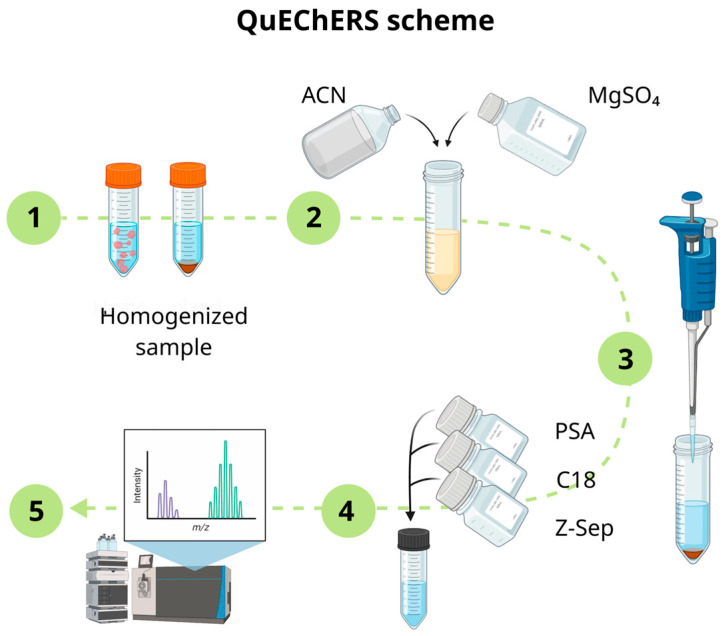
Schematic representation of the QuEChERS method for the extraction and purification of mycotoxins. Homogenized sample aliquots (1) are extracted with acetonitrile (ACN), followed by the addition of salts (MgSO_4_, NaCl, or citrate buffers) to induce phase separation via a salting-out effect (2). The organic phase (3), containing the target analytes, is subjected to additional cleanup by dispersive solid-phase extraction (d-SPE) employing sorbents such as PSA, C18, and Z-Sep to remove lipids, sugars, pigments, and organic acids (4). The resulting purified extract is analyzed using LC–MS/MS, HPLC–FLD, or UHPLC–HRMS (5).

The QuEChERS method (Quick, Easy, Cheap, Effective, Rugged, and Safe) was originally developed as a universal procedure for the extraction and purification of pesticides from food products. It was subsequently adapted for mycotoxin analysis. QuEChERS is a combined sample preparation approach that enables the simultaneous extraction and cleanup of a broad range of mycotoxins. The principle involves extracting a sample (typically a homogenized food matrix) with an organic solvent, followed by the addition of salts (e.g., MgSO_4_, NaCl, and sometimes citrate buffers) to induce phase separation via a salting-out effect. Under these conditions, the organic phase separates from the aqueous phase and solid residues, concentrating the target analytes. Further cleanup is achieved by dispersive solid-phase extraction (d-SPE), wherein sorbents such as PSA, C18, Z-Sep, MgSO_4_, and others are added to remove lipids, sugars, organic acids, and pigments [48]. The purified extract is subsequently analyzed using LC-MS/MS, HPLC-FLD, or UHPLC-HRMS. The recovery and specificity of this method are comparable to those achieved with immunoaffinity columns [49] (Figure 7). Its main limitation is that residual matrix effects are often incompletely removed, so additional cleanup is frequently required.

### 3.2. Methods Based on Chromatographic Separation

This group comprises methods in which the separation of mycotoxins occurs due to differences in their interactions with the mobile and stationary phases. These techniques enable high selectivity and resolution. Chromatographic methods are regarded as the most accurate and reproducible; however, they require expensive instrumentation and meticulous optimization of operating conditions. Unlike SPE, which shares the same analyte–sorbent principle, chromatography separates analytes dynamically in a flowing phase, with selectivity tuned through the mobile phase and sorbent, and it provides not only purification but also qualitative and quantitative determination.

#### 3.2.1. Silica Gel Column Chromatography/Flash Chromatography

Column chromatography—and its accelerated variant, flash chromatography—is a separation technique based on differential retention of mixture components on a stationary phase as a mobile phase flows through a column. The stationary phase typically consists of silica gel (SiO_2_) with defined activity and particle size. The mobile phase is selected as a solvent mixture of variable polarity (applied in gradient or stepwise mode; e.g., CHCl_3_–MeOH, 90:10, for the trichothecene mycotoxin from Fusarium [50]) such that individual components exhibit distinct affinities for the stationary phase. As a result, more polar substances—those with stronger affinity for silica gel groups—are retained within the column. Mycotoxin molecules possessing such affinity interact with the adsorbent surface via polar silanol groups through hydrogen bonding, dipole–dipole interactions, or, in the presence of aromatic rings, π-bonding. Less polar compounds or those more soluble in the mobile phase migrate through the column more rapidly.

In flash column chromatography, pressure (typically atmospheric pressure with slight gas overpressure—air or nitrogen) is applied to accelerate the flow of the mobile phase, substantially reducing analysis time. Two-step silica gel column chromatography is often employed to achieve satisfactory separation [51]. However, silica gel column chromatography is not always effective for isolating mycotoxins from complex matrices, such as cereal-based samples in the determination of zearalenone [52].

#### 3.2.2. High-Performance Liquid Chromatography

High-performance liquid chromatography (HPLC) is the most widely used and one of the most accurate classical methods for achieving analytical-grade purity. It is commonly coupled with UV, fluorescence, amperometric, or spectrofluorimetric detection. Like the techniques described above, HPLC relies on differential interactions of mycotoxin molecules with the stationary and mobile phases. While analytical HPLC is employed for the quantitative determination of mycotoxins, preparative HPLC (prep-HPLC) can be used for their further purification. Reversed-phase sorbents, particularly C18 and C8, are most frequently applied for mycotoxin separation due to their capacity to resolve a broad range of these compounds effectively. When necessary, ion-exchange or specialized sorbents may be utilized, particularly if the toxin bears charged or aromatic moieties. HPLC is often integrated with preliminary cleanup steps to enhance performance [53].

#### 3.2.3. Gas Chromatography

Gas chromatography is employed for the analysis of volatile and thermally stable compounds. Since the majority of mycotoxins are non-volatile and thermolabile, they require derivatization prior to analysis. In this process, specific functional groups of the analytes are converted into derivatives with altered reactivity, solubility, boiling point, melting point, physical state, or chemical composition—for instance, transformation into trimethylsilyl derivatives [54]. Following derivatization, mycotoxins can be introduced into the GC column and separated on the stationary phase. However, this method is associated with certain limitations, as it necessitates multi-step sample preparation, including hydrolysis, cleanup, and derivatization prior to analysis [55].

### 3.3. Methods Based on Liquid–Liquid Distribution

These methods are based on differences in the partition coefficients of mycotoxins between two immiscible liquid phases, typically aqueous and organic. Unlike sorption-based techniques, no solid phase is involved, thereby eliminating the risk of irreversible adsorption or degradation of thermolabile compounds. Such approaches are employed for gentle purification and preparative separation of mycotoxins while preserving their structural integrity, as exemplified by liquid–liquid extraction (LLE) discussed previously. The main drawbacks of these methods include the complexity of system optimization and phase selection, as well as relatively low throughput compared to modern chromatographic techniques.

#### Countercurrent Chromatography (CCC, CPC)

High-speed countercurrent chromatography (HSCCC) and centrifugal partition chromatography (CPC) are liquid–liquid chromatographic techniques in which both the stationary and mobile phases are liquids. The method relies on centrifugal force to retain one phase (the stationary phase) within the column, while the mobile phase is continuously pumped through it. This enables separation based solely on differential partitioning between two immiscible liquid phases, without any solid support. This approach has demonstrated particular efficacy in the isolation of patulin [56]. CCC is especially suitable for labile compounds such as trichothecenes, as it minimizes analyte loss caused by adsorption and irreversible interactions commonly associated with solid stationary phases [57]. A key limitation of countercurrent chromatography lies in its reduced resolving power for structurally similar compounds. Separation of closely related mycotoxins may require multiple runs, modification of the biphasic solvent system, or combination with complementary techniques—including preliminary cleanup steps such as resin-based fractionation [58].

### 3.4. The Need for Novel Methods and Solutions

Classical methods for sample preparation and separation of mycotoxins remain essential tools for analysts today. Each has demonstrated its effectiveness and has been incorporated into numerous studies and standardized protocols. However, all of these approaches possess inherent limitations that complicate their application in routine analysis, particularly in multicomponent contexts. Their principal characteristics and limitations are compared in Table 2.

These challenges stem largely from the diverse and complex chemical structures of mycotoxins. As a result, it is virtually impossible to select a single universal stationary phase or a unified extraction protocol—some toxins are efficiently recovered, while others are partially or completely lost.

Moreover, the sample matrix itself contains a vast array of co-extracted substances—including lipids, sugars, proteins, pigments, and organic acids—that interfere with extraction, contribute to matrix effects in LC–MS/MS analysis, compete for sorbent binding sites, or participate in side reactions during derivatization (as in GC).

Many methods necessitate a trade-off between selectivity and broad-spectrum applicability. Ion-exchange and immunoaffinity columns, for example, offer high selectivity but are limited to a narrow range of toxins. QuEChERS is versatile and rapid, yet it often fails to eliminate matrix effects and frequently requires additional cleanup steps. Gas chromatography enables detection of specific mycotoxin classes but relies on derivatization and is unsuitable for thermolabile compounds.

Thus, the simultaneous isolation and separation of a wide spectrum of mycotoxins remains an unresolved challenge. The singular application of any traditional method—without combination with other techniques—fails to achieve an ideal balance of sensitivity, selectivity, simplicity, and universality. These constraints underscore the urgent need for novel strategies and solutions. Emerging technologies are increasingly directed toward improving cleanup efficiency, minimizing matrix effects, and enhancing the reliability and reproducibility of multi-mycotoxin analysis.

## 4. Promising Methods for the Separation of Mycotoxins in the Extract

Whereas the classical methods above were developed largely by empirical optimization, an alternative is to build selectivity into the isolation step itself by exploiting specific molecular interactions. This section surveys four emerging strategies aligned with this idea: directed co-crystallization, supercritical-fluid-assisted crystallization, smart MOF/COF membranes, and physics-informed machine learning. These strategies differ markedly in maturity. Co-crystallization and supercritical-fluid extraction are well established for pharmaceuticals and other natural products, whereas the membrane and machine-learning concepts are presented here as forward-looking proposals whose application to mycotoxins remains to be demonstrated. We therefore treat this section as a perspective on how precision mycotoxin isolation might develop, indicating in each case what is already supported by experimental evidence and what is still conceptual.

### 4.1. Cocrystallization

Cocrystallization is a process involving the formation of multicomponent crystalline materials through the interaction of a target compound with a coformer via non-covalent bonds. Unlike conventional separation methods, which rely on differences in polarity or phase distribution, cocrystallization exploits molecular selectivity: the coformer interacts exclusively with compounds capable of forming stable supramolecular structures. This enables the separation of substance mixtures at the crystallization stage—the target compound is isolated as a cocrystal, while impurities remain in solution. This mechanism renders cocrystallization a promising tool for the isolation of mycotoxins following extraction, particularly in cases where compounds exhibit similar polarity, structural analogy, or exist as isomers that are poorly resolved by classical techniques.

The selection of suitable coformers is a critical step in the design of cocrystal systems. The formation of a stable cocrystal depends on complementary molecular recognition between the active ingredient and the coformer, often facilitated by hydrogen bonding, π–π stacking, or halogen bonding. Computational screening methods are increasingly employed alongside experimental techniques to identify compatible coformer candidates. An ideal coformer should exhibit sufficient supramolecular synergy with the active ingredient while changing physicochemical properties such as solubility or photostability.

**Figure 8:**
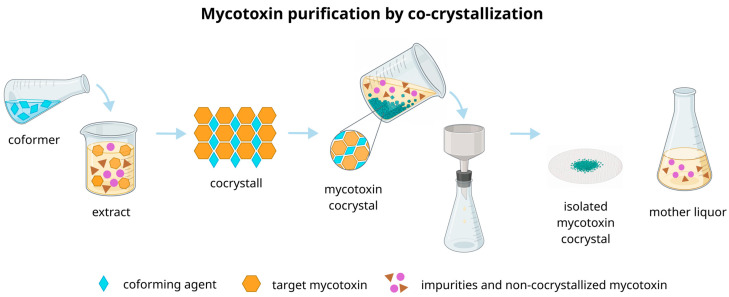
Schematic representation of mycotoxin purification via cocrystallization. The coformer added to the extract interacts exclusively with the target mycotoxin, forming a stable cocrystal that precipitates. Unreacted impurities and cocrystallization by-products remain in solution and are subsequently removed.

Cocrystallization is now recognized as a fully-fledged method for the highly selective separation and purification of organic compounds, including difficult-to-resolve chiral pairs and mixtures of structural analogues—an approach that may be extended to mycotoxins. In several fields, such as pharmaceuticals and food science, the technique has been successfully employed both for the isolation of target compounds via the formation of their cocrystals (1) and for the removal of impurities through the selective cocrystallization of unwanted components (2). In both scenarios, a coforming agent is selected that forms a cocrystal exclusively with either the target analyte or the impurity (Figure 8). Despite the presence of structurally similar compounds and the inclusion of impurity species into the crystal lattice of the cocrystal, the resulting systems exhibit improved physicochemical properties. The separation of such crystalline forms is readily scalable and can be rendered highly efficient through rational design—including the construction of solubility phase diagrams, optimization of solvent systems, and selection of appropriate cocrystallization methods [59]. Thus, the technology has already demonstrated its effectiveness across a broad range of organic compounds and holds substantial potential for adaptation to the selective purification and separation of mycotoxins.

One of the most extensively developed applications of cocrystallization is in chiral separation. For example, praziquantel (PZQ)—an important chiral antiparasitic drug commercially available as a racemate—was purified via two-step cocrystallization with L-malic acid (L-MA). Owing to the absence of acid–base functional groups, chiral resolution of RS-PZQ cannot be achieved through salt formation, and alternative methods have not proven industrially viable. The resulting R-PZQ:L-MA cocrystals exhibited improved solubility compared to both the enantiomerically pure form of the drug and the racemate [60]. Another example of chiral resolution involves the separation of ofloxacin using tartaric acid derivatives [61]. The authors demonstrated that D-DBTA and L-DBTA selectively form cocrystals with the corresponding enantiomers of ofloxacin in aqueous media, highlighting the environmental compatibility and high selectivity of the method. In a seminal study [62], cocrystallization was employed for the first time to resolve a non-salt-forming racemate: enantiospecific hydrogen bonding between the target compound and S-mandelic acid enabled selective precipitation of only one enantiomer, achieving up to 70% recovery of the desired enantiomer in a single crystallization cycle. Racemic halogenated mandelic acids have also been resolved through the formation of enantioselective cocrystals with levetiracetam [63]. Notably, the position and type of halogen substituent significantly influenced selectivity, underscoring the critical role of molecular complementarity in cocrystal design. Dehydrocholic acid (DHA) was employed for the resolution of arylmethyl sulfoxides [64], forming cocrystals with the R- and S-enantiomers of p-XC_6_H_4_SOMe (X = Me, OMe, Br, H). The resulting solid phases were mutually isomorphic yet structurally distinguishable.

To develop suitable chiral resolution processes, the classification of solid forms (conglomerate, racemic compound, diastereomeric pair) and the construction of pseudobinary phase diagrams for cocrystals formed between chiral target compounds and potential coformers in a given solvent system can be employed [65]. To accelerate screening, systematic structural modification of a successful coformer scaffold may also be applied—for example, cocrystallization of the aforementioned praziquantel (PZQ) with coformers analogous to malic acid, including tartaric, methylsuccinic, 2,3-dimethylsuccinic, and 2,2-dimethylsuccinic acids [65].

Cocrystallization can also serve as an effective strategy for the isolation and simultaneous property modification of natural biomolecules. Dihydromyricetin (DMY) from vine tea was purified via cocrystallization, yielding both the pure compound and its cocrystalline forms. All three solid forms retained high antioxidant activity, as confirmed by DPPH assay; however, the cocrystals exhibited enhanced antibacterial activity against drug-resistant CRAB and MRSA strains compared to the pure compound [66].

An equally important direction is the formation of cocrystals during the extraction process, which could be particularly significant for handling natural toxins. The study [67] revealed that tropane alkaloids and the ionic liquid [C3tr][PF6], being structurally similar, are capable of forming a cocrystal upon cooling of the extract. This demonstrates that structural complementarity between the solvent and the substrate can not only enhance extraction selectivity but also initiate the formation of a separable crystalline phase, significantly simplifying purification. Such an approach could be extended to systems containing mycotoxins.

Unlike separation methods based on the host–guest mechanism, where the target molecule is incorporated into the cavity of a host structure and, after the separation process, necessarily requires complex dissociation and an additional isolation step, cocrystallization forms a new crystalline phase. Cocrystals are characterized by strict stoichiometry and are thermodynamically independent solid phases, rather than inclusion complexes [68]. This renders cocrystallization more technologically convenient: the obtained crystalline forms can be used directly, without dissociation, while also possessing improved physicochemical properties compared to the original substance.

An additional advantage of cocrystallization compared to classical approaches lies in the enantiospecificity of directed intermolecular interactions. The formation of cocrystals is governed by differences in the interaction energy between the coformer and each enantiomer, leading to the formation of diastereomeric solid phases with comparable crystal structures but differing solubility or melting points. This enables their efficient separation via simple recrystallization. For some structurally similar isomeric forms of mycotoxins, separation remains a challenging analytical task. For instance, even the enantiomeric isomers of trichothecenes, 3-ADON and 15-ADON, exhibit near-complete indistinguishability on standard chromatographic sorbents and require the use of chiral stationary phases for selective separation [69,70]. In contrast to such methods, cocrystallization combines the processes of separation and property modification of the substance into a single step, making it a promising and scalable tool for the purification and potential chiral (or isomer-specific) separation of mycotoxins [71,72,73,74,75,76].

### 4.2. Separation Using Supercritical Fluids

In most cases, the target mycotoxin molecule does not crystallize from the extract due to its high solubility in the given solvent. However, in some instances, such a target molecule may fail to crystallize because of a number of factors hindering the nucleation of the crystalline phase. One such factor is the “competition” between different conformations of the target molecule, which interfere with each other. In solution, molecules can adopt various conformations, which directly affects whether they will crystallize and, if so, which polymorph will form, thereby influencing the driving force of crystallization. One method enabling the isolation of mycotoxins is supercritical fluid extraction.

Supercritical fluid extraction is based on the phenomenon of the supercritical state of matter. At temperatures and pressures above the critical point, the densities of the liquid and gas phases of a substance coincide, and the substance forms a supercritical fluid (SCF). Substances that exist as SCFs include carbon dioxide, water, ammonia, methane, and others. Supercritical fluids possess unique properties—low reactivity and negligible surface tension—which make them excellent “green” solvents. Due to its high density, a supercritical fluid dissolves substances (like a liquid), and owing to its low viscosity and high diffusivity, it easily penetrates the matrix (like a gas). Mass transfer of the substance from the solid phase into the flow of the supercritical extractant occurs, which then passes into a separator. In the separator, the pressure and temperature are reduced, the solvent ceases to be effective, and the obtained analytes are precipitated. Modification of the SCF with polar co-solvents (ethanol, methanol) increases the selectivity of the method. Furthermore, due to the unique properties of the supercritical fluid, extraction requires significantly less time and resources than classical liquid–liquid extraction and rivals chromatographic methods due to its low cost and environmental friendliness. Moreover, by manipulating the pressure of the SCF, its solvating power can be influenced, which is of great interest for the isolation of crystalline phases using fluids. SCF has indeed been applied to mycotoxins, principally aflatoxins and, more recently, ochratoxin A and emerging toxins in food and feed matrices [77,78,79].

However, a fundamental challenge of the method often remains in the background: its limited inherent selectivity. A supercritical fluid, especially in combination with polar modifiers (methanol, ethanol, water), represents a powerful but “crude” tool. Its ability to dissolve the target mycotoxin is almost always accompanied by the parallel extraction of a wide range of accompanying substances. Consequently, the researcher obtains not a purified toxin, but a new, often more complex mixture where the target analyte is dissolved in a “cocktail” of compounds of similar polarity and molecular weight extracted from the initial matrix—SCF distinguishes substances by their physicochemical parameters, not by their chemical identity. Furthermore, the issue of stereoisomers, enantiomers, and compounds with similar chemical structures (mycotoxin families often have similar chemical structures, e.g., fumonisins) remains relevant. Although supercritical fluid is used for the extraction of substances, it does so in a non-targeted and insufficiently selective manner, leading to the extraction of a set of substances that require further separation.

**Figure 9:**
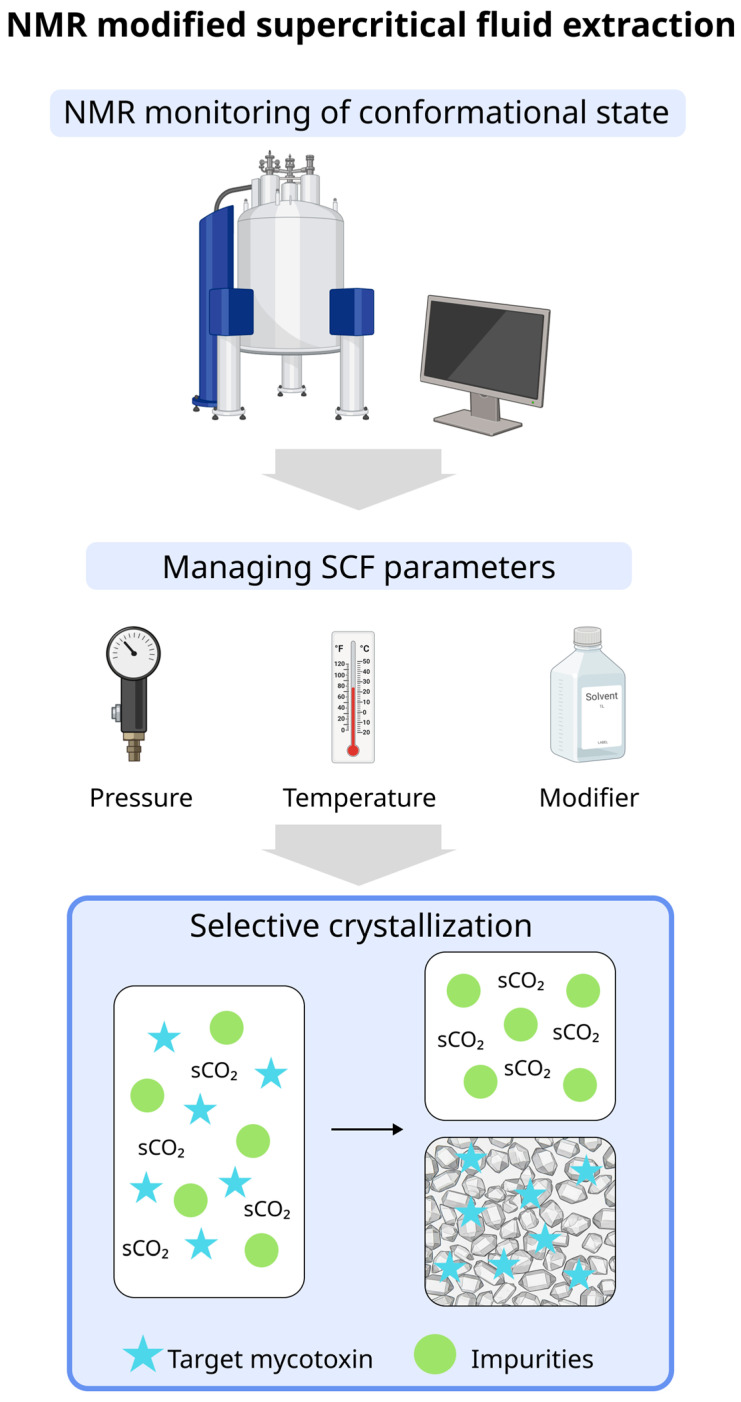
Selective Crystallization Using Supercritical Fluids (SCF) and NMR Spectroscopy.

It is evident that a necessary “fine-tuning” of this method is required. NMR spectroscopy can serve as a “sight” for this purpose. NMR spectroscopy allows for the assessment of the conformational state of all molecules in the extract and can predict which substances will crystallize and which will remain in solution. Hydrogen bonds, which constitute the primary molecular interactions during crystal formation, can be effectively studied as a function of pressure and temperature in a supercritical fluid solvent (Figure 9) [80]. NMR under supercritical conditions enables the determination of the conformer distribution of ibuprofen in supercritical CO_2_, with results consistent with molecular dynamics models [81]. SCF technology is also used for the controlled formation of solid forms, for example, bicalutamide [82]. Transferring the existing knowledge to the challenge of mycotoxin isolation, the following combination of methods can be proposed: first, the conformational state of the target molecule is determined using NMR; subsequently, the optimal supercritical fluid conditions are selected, under which only the target mycotoxin crystallizes, while by-products remain dissolved in the fluid. This approach is promising as it allows for increased extraction selectivity while simultaneously reducing the need for subsequent purification steps, opening up avenues for the highly efficient and “green” isolation of mycotoxins.

### 4.3. Cybernetic Membrane

In recent years, high-performance framework structures—porous solid materials capable of molecular recognition via the host–guest principle—have found increasingly broad application in the field of separation [83,84,85]. Key among these are metal–organic frameworks (MOFs) [86] and covalent organic frameworks (COFs) [87]. Owing to their record-high specific surface areas, regular porous architectures, and the ability to finely tune pore chemistry, these materials demonstrate impressive selectivity in model systems. However, their effectiveness declines sharply when transitioning to real-world natural extracts.

MOFs are hybrid crystalline structures composed of inorganic nodes (metal ions or clusters) connected by organic linker ligands. This architecture enables the formation of a regular system of micropores with a highly developed surface, rendering MOFs exceptionally efficient adsorbents. COFs, in contrast, are formed exclusively from organic building blocks linked by strong covalent bonds, which endows them with high chemical and thermal stability.

Despite these advantages, the vast majority of studies on porous membranes are conducted on relatively simple model mixtures with controlled compositions. When applied to real-world objects, such as fungal extracts, a number of fundamental challenges arise, stemming from the complexity and variability of natural matrices.

First, mycotoxins in natural extracts are never present as simple binary or ternary mixtures. A real sample contains dozens of accompanying metabolites, pigments, lipids, and other products of fungal metabolism. Under membrane separation conditions, this leads to partial pore blockage, nonspecific adsorption of impurities, reduced permeability, and, ultimately, a loss of selectivity.

Second, the composition of extracts is highly variable, depending on the producer strain, cultivation conditions, and sample preparation method. A membrane optimized for a fixed composition (e.g., for a specific set of mycotoxins) proves ineffective when that composition changes. The adaptive properties of MOFs, such as the framework “breathing,” are only partially effective in this context: they respond to integral environmental parameters (temperature, solvent nature) but are incapable of differentially responding to the appearance of a specific class of undesirable impurities.

Thus, modern MOF- and COF-based membranes, despite being “smart” in their structure, remain functionally passive. Because they receive no information about what is occurring in the separated stream, they cannot purposefully adapt to changes in its composition. This limitation is not technical but methodological in nature and necessitates a fundamental revision of the very concept of membrane separation.

**Figure 10:**
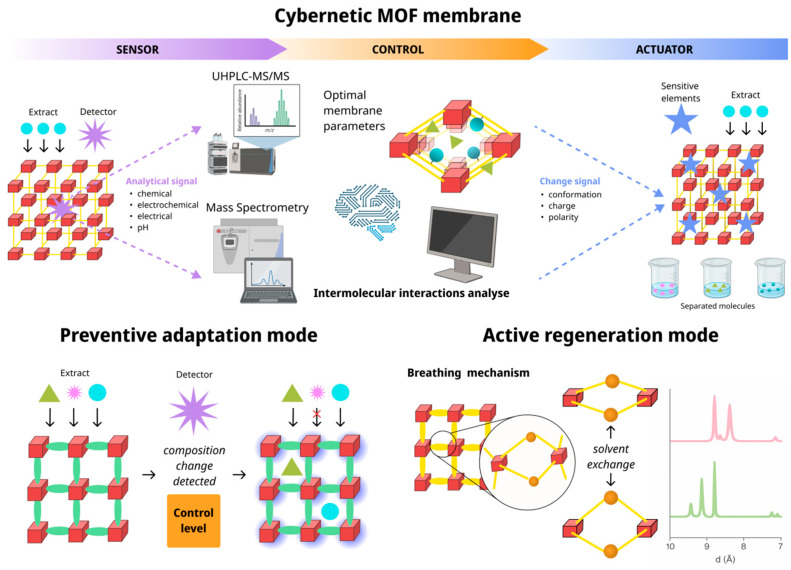
Schematic representation of the cybernetic membrane.

We propose the cybernetic membrane as a long-term vision for the field rather than a near-term, deployable design: a hybrid system in which selective transport is inseparably linked to medium analysis and controlled reconfiguration of the pore space. Such a system would implement closed-loop (feedback) control, in principle enabling deterministic selectivity under a priori compositional uncertainty (Figure 10). No feedback-controlled membrane of this kind has yet been reported for mycotoxin isolation; we therefore present it as a target architecture, not a validated method.

A cybernetic membrane comprises three functional levels integrated into a single technological module:

- Sensor level (diagnostics)—provides continuous data acquisition on the properties of the separated medium at the membrane inlet or directly within the pore space.
- Control level (decision-making)—processes signals from the sensors, identifies changes in the “molecular pattern,” and selects the optimal membrane operating mode based on a library of precise data on intermolecular interactions.
- Actuator level (execution)—implements the selected scenario by generating an external stimulus that initiates reversible reconfiguration of the porous framework.

The framework material itself (MOF or COF) serves as the actuating element; however, its structure must incorporate built-in mechanisms for controlled property modulation.

At the sensory level, various environmental monitoring methods can be employed, such as mass spectrometry [88] or its combination with UHPLC [89]. This is not fundamentally novel or unfeasible. Subsequently, a signal (chemical, electrochemical, electrical, medium acidity, etc [90].) is transmitted to the membranes themselves. For this purpose, sensitive elements must be introduced into the membrane structure—fragments that reversibly change their conformation, charge, or polarity under the influence of external stimuli. Examples include thermosensitive linkers [91,92,93], electrosensitive frameworks [94,95,96], photochromic ligands [97,98], and pH-switchable frameworks [99,100,101].

Two strategies are then possible:

(1) Preventive adaptation mode. Sensors placed at the inlet of the membrane module detect changes in the composition of the flow (e.g., an increase in lipid or pigment concentration) before these components reach the membrane. The control unit initiates a framework reconfiguration (e.g., closing large pores or increasing surface hydrophobicity), thereby preventing fouling and loss of selectivity.
(2) Active regeneration mode. Sensors located directly within the pore space detect the onset of nonspecific sorption (e.g., via molecular rotors or QCM). The system applies a short-term stimulus (thermal pulse, potential change) that induces a “breathing” motion of the framework. This leads to the desorption of weakly bound “interloper” molecules and restores permeability without process interruption.

In the current paradigm of MOF/COF utilization, their unique properties, stimulus response, and “breathing” frameworks are not used to their full potential. In a cybernetic system, the membrane receives information about the environment through the sensory level and selects the type of response that is optimal for the current situation. This transforms it from a passive filter into an active molecular interface, whose selectivity is not merely embedded during synthesis but is dynamically maintained during operation based on feedback data.

The key advantage of this approach is its functionality under conditions of fundamental information incompleteness. We do not need to know the exact composition of the extract or predict all possible variations. It is sufficient to detect integral parameters (the appearance of classes of compounds with specific properties) and to have a library of responses capable of neutralizing typical threats—pore blocking, nonspecific sorption, competitive displacement.

Further development of this direction will require solving a number of engineering challenges: the design of stable and fast sensory elements compatible with chemically aggressive environments; the creation of reliable methods for local delivery of stimuli to the framework; and the miniaturization of control electronics. A less ambitious near-term step toward this vision avoids the full sensor–control–actuator loop. Many mycotoxins are weak acids, weak bases, or ampholytes, so their net charge is set by medium pH (Figure 4). A pH-responsive MOF/COF membrane could switch a single target between retention and release within a complex extract. During loading, the membrane is set to a state that binds the protonated or deprotonated analyte. A defined pH change then triggers its selective release, while differently charged matrix components stay bound or are washed through. pH-switchable frameworks of this type are already documented [102,103]. This single-stimulus, open-loop variant is therefore a realistic first objective. It offers a natural bridge from today’s passive membranes toward the fully cybernetic system above. Even so, the direction is clear: separation systems that do not merely filter but recognize, analyze, and adapt. We present the cybernetic membrane as a long-term goal rather than a method ready for mycotoxin work today; its role here is to indicate where MOF/COF separation could eventually lead—toward purification interfaces whose selectivity is maintained by feedback rather than by adsorption statistics alone—once the sensing, actuation, and control problems above are solved.

### 4.4. Physics-Integrated AI

The use of such technologies as described above is a complex challenge, one that will undoubtedly be realized in the future. However, at present, approaches related to the integration of AI have developed so broadly and deeply that such tasks are not fundamentally unsolvable [104,105,106,107].

The approaches outlined above—directed cocrystallization, supercritical-fluid-assisted crystallization, and adaptive MOF/COF membrane concepts—share a common bottleneck: choosing the correct resolving environment (coformer, solvent/modifier, pressure–temperature window, pore chemistry, stimulus protocol) under conditions where the extract composition is complex, variable, and partially unknown. Traditionally, this choice is made by iterative experimental screening, which is slow, expensive, and poorly transferable between laboratories or matrices. In multi-mycotoxin contexts, brute-force screening becomes essentially combinatorial: dozens of target analytes, hundreds of potential coformers or framework chemistries, multiple solvents, and many operating regimes.

At the same time, the past decade has produced a qualitative shift in what can be predicted with data-driven models—especially when machine learning is not used as a “black box” but coupled to molecular representations that encode the relevant physics. Therefore, we propose a predictive paradigm: combine machine learning with a physics-based representation to identify resolving agents and operating windows for selective separation of mycotoxins. In this framework, AI does not replace chemical understanding; it formalizes and scales it.

This is more than aspirational. Machine learning has already proven effective at optimizing separations directly: reinforcement learning has been trained to design liquid-chromatography gradients autonomously, with the trained agent clearly outperforming both random and conventional gradient programs [108], and closed-loop Bayesian optimization, interfaced directly with the chromatograph, has developed satisfactory gradient methods for highly complex (n > 80) mixtures in only a few tens of experiments [109]. These results show that data-driven optimization can navigate the large, high-dimensional condition space that makes separation development slow—precisely the obstacle facing the precision-extraction strategies considered here.

However, a purely empirical model trained on “success/failure of crystallization” is usually brittle because crystallization outcomes depend on subtle, context-specific interactions: hydrogen-bond competition, ionization state, solvation, conformational flexibility, and impurity effects. For extracts, the situation is even more complex because the matrix perturbs the effective chemical space.

Hence, the core idea is to encode chemical physics into the input representation. Instead of learning directly from SMILES strings alone, we propose a hybrid representation consisting of the following: molecular graph + 3D conformers (to capture shape complementarity and conformational competition); supramolecular interaction fingerprints (donor/acceptor patterns, halogen-bond motifs, aromatic stacking propensity, cation–π likelihood); ionization-aware descriptors (pK_a_, microstate distribution vs. pH, salt-forming likelihood); solvation descriptors (Hansen parameters, COSMO-like polarity measures, H-bonding capacity of solvents/modifiers); competitive binding features that approximate “matrix pressure” (e.g., abundance-weighted classes: lipids/proteins/phenolics/organic acids) [110,111,112,113]. This is particularly relevant for mycotoxins, where small differences in functional group placement (e.g., acetylation position in trichothecenes) can dramatically alter intermolecular recognition while remaining chromatographically hard to resolve.

We envision the learning problem as a “molecular pairing” task: given a target molecule (mycotoxin) and a candidate resolving agent (coformer, ionic liquid, MOF/COF fragment/linker motif), predict the probability that the pair produces a useful separation outcome under specified conditions.

A transformer model is a natural choice because [114,115,116]:

- It can learn context-dependent interactions (the same functional group behaves differently depending on its neighborhood);
- It can integrate multiple modalities (sequence/graph tokens + continuous descriptors);
- It supports transfer learning from large chemical corpora.

Moreover, a large amount of data has accumulated in the literature on which such models can be trained [116,117].

To enable proper training of AI models, we recommend that authors perform a number of simple steps in their experiments, which will help future AI specialists develop models regardless of the architecture used:

(1) Define the target analytes unambiguously. List full names and abbreviations for all mycotoxins. If standards are used, report supplier, stated purity, exact chemical form, and storage conditions if relevant.
(2) Clearly indicate the experimental context by specifying whether neat standards, spiked matrices, or real extracts were used. For neat standards, report the solvent system and purity; for spiked matrices, state whether spiking was performed before extraction (to evaluate overall method performance) or after extraction (to assess matrix effects and post-extraction steps); for real extracts, describe the biological source in detail, including the producer strain or species, substrate or commodity (e.g., wheat grain, liquid medium), and relevant cultivation or storage conditions (e.g., temperature, incubation time, water activity). This ensures clarity, reproducibility, and proper interpretation of the results.
(3) Report mixture composition and intended competitors. If multi-mycotoxin mixtures are studied, list which toxins were present together and their approximate concentration ranges/ratios. Report any intentionally added interferents/surrogates.
(4) Describe the sample matrix in chemically meaningful terms. Provide matrix identity (commodity/type), physical state (raw/processed), sample mass used, and key descriptors where available (moisture %, fat content or at least “high-fat”, pH). Include pretreatment (grinding/sieving/drying) and storage (time, temperature, humidity if controlled).
(5) Write the full protocol as an engineering recipe, not a reference citation. For every step, provide exact solvent composition, measured pH (when applicable), solid-to-liquid ratio, times, temperatures, agitation conditions, and phase-separation details (centrifugation RCF/time; filtration type/pore size).
(6) Specify all materials with traceability. For sorbents, membranes, cartridges, and consumables: type, manufacturer, catalog/batch if possible, mass/bed volume, dimensions. For in-house materials (MOF/COF), provide full synthesis (or a complete reference) plus characterization confirming identity and activation state.
(7) Account for volumes and mass balances through the workflow. Report collected volumes, evaporation conditions, reconstitution solvent and final volume, split ratios, and any dilution/concentration steps. This is essential for reproducible recovery calculations.
(8) Provide analytical method details sufficient for reproduction and comparison. Report instrument type, column and LC gradient (or GC thermal program/derivatization), ionization mode and key transitions (LC–MS/MS), internal standards, calibration strategy, and identification/quantification criteria.
(9) Report quantitative performance metrics explicitly. For each analyte (or a clearly justified representative set): recovery (%) with n and SD/RSD at ≥2 levels where possible; matrix effect with the exact formula used; LOD/LOQ and how defined/calculated.
(10) Document selectivity evidence, not only “it works.” Provide chromatograms/spectra or fraction-composition data demonstrating separation and impurity reduction. For flow systems, provide time/volume-resolved performance (e.g., breakthrough-type data) when applicable.
(11) Report failures and limitations with chemical context. Specify which analytes fail, where losses occur (which step), and under what conditions (pH range, high-fat matrices, water content, sorbent overload, emulsions, fouling, non-reproducible crystallization, phase transformations). Boundary conditions are valuable scientific output.
(12) Use Supplementary Information to provide reusable primary outputs. Include (i) a single protocol table (steps, solvents, volumes, times, pH, temperature), (ii) compound list table (targets, standards, coformers), (iii) calibration ranges/equations, and (iv) primary raw/near-raw outputs when feasible (peak area tables; PXRD/DSC/FTIR raw files or peak lists). No ML formatting is required.

Critically, the success of this approach depends on the availability of high-quality, richly annotated experimental data. The twelve recommendations provided above are not merely editorial suggestions; they constitute a minimal reporting standard that can make published results machine-actionable and reusable for training. We deliberately avoid synthetic data, emphasizing instead the wealth of existing and future literature data—provided it is reported with sufficient chemical and procedural detail.

Looking forward, the convergence of automated experimentation (high-throughput crystallization, robotic screening), in situ characterization (real-time feedback on crystal form and purity), and the predictive models described here will likely enable self-optimizing separation systems. Such systems would not only recommend candidate resolving agents but also adapt to matrix variability on the fly—a step toward the cybernetic membrane concepts discussed earlier in this chapter.

We therefore invite the community to adopt these reporting practices and to contribute to open, curated datasets. The tools for a revolution in separation science are already within reach; what remains is the collective effort to build the knowledge infrastructure that will drive it.

## 5. Conclusions

Thus, despite significant progress, existing isolation methods often remain either insufficiently selective, necessitating multi-step purification, or unsuitable for thermolabile or low-concentration target compounds. This underscores the need for a fundamental paradigm shift in problem-solving approaches.

Rather than relying on an empirical search for conditions under which a target substance separates from impurities, a transition toward precision methods with deterministic control is required. The objective is to engineer the process a priori, ensuring that unwanted interactions are minimized and selectivity is maximized at each step, based on a comprehensive understanding of the molecular properties of both the target compound and the impurities.

This review examines approaches unified by the philosophy of rational design and molecular engineering. The methods discussed—cocrystallization, supercritical fluid extraction, MOF/COF-based membranes, and computational prediction for ‘precision’ or ‘targeted’ extraction, in which predictive modeling, rather than chance the pivotal role. What unites these methods is their ability to orchestrate the isolation process at the level of molecular interactions. This ensures high product purity and predictable yields while minimizing by-product formation.

At the same time, these strategies warrant appropriate caution. They differ markedly in maturity, and none currently represents a validated, turnkey solution for mycotoxin isolation. Cocrystallization and supercritical fluid extraction are well established for pharmaceuticals and other natural products, yet their selective application to mycotoxins—especially to closely related isomers within real fungal extracts—still awaits systematic experimental confirmation. The cybernetic membrane and physics-integrated AI concepts remain, at present, largely conceptual: their realization depends on unresolved engineering challenges (robust in-line sensing, reversible framework actuation, and miniaturized control) and on the accumulation of high-quality, richly annotated datasets. Each approach also carries intrinsic constraints—the dependence of cocrystallization on a suitable coformer, the limited inherent selectivity of supercritical fluids, the fouling and batch-to-batch variability of porous membranes in complex matrices, and the data requirements and limited transferability of machine-learning models. We therefore regard precision extraction not as a ready-made replacement for classical techniques, but as a complementary and still-developing direction. Its realization for mycotoxins will require focused experimental validation, transparent and standardized reporting, and integration with the established methods that remain indispensable today.

## Figures and Tables

**Table 1:** Polarity of selected mycotoxins. This table presents several mycotoxins and a characterization of their polarity. For the extraction of relatively polar trichothecene mycotoxins, including verrucarol (VOL), diacetoxyscirpenol (DAS), and deoxynivalenol (DON), ethyl acetate or diethyl ether are used [29]. Mid-polar aflatoxin B1 is most efficiently extracted using chloroform [30]. Lipophilic trichothecene toxins T-2 and HT-2 have been extracted with ethyl acetate [31,32].

Mycotoxin	Molecule	LogP	Functional Groups	Suitable Solvent
Vomitoxin (polar)	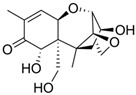	−0.71	Multiple hydroxyl groups and a carbonyl group	Ethyl acetate, diethyl ether
Aflatoxin B1 (mid-polar)	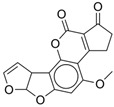	1.23	Lactone groups, keto-type carbonyl groups (C=O); epoxy bridge (oxygen three-membered group)	Ethyl acetate, dichloromethane, chloroform
T_2_ toxin (non-polar)	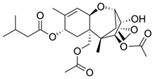	2.27	Epoxy group, ester groups	Dichloromethane, chloroform, ethyl acetate, and hexane (for lipid removal when necessary)

**Table 2:** Principal strengths and limitations of the classical mycotoxin extraction and separation methods.

Method	Strengths	Key Limitations
LLE	Simple and universal; minimal equipment	Process intensive and solvent-heavy; emulsions and weak cleanup cause losses
SPE	Established cleanup before LC–MS/MS	No single sorbent suits all toxins; IAC is costly and lasts only 1–3 runs
d-SPE/m-SPE	Fast dispersive cleanup	Must be re-optimised for each new matrix
QuEChERS	Quick, cheap, broad multi-toxin coverage	Matrix effects often persist; cleans up but does not separate
Column/flash chromatography	Accessible preparative fractionation	Weak for complex matrices and close isomers; often two-step
HPLC/prep-HPLC	Highest resolution; suits both analysis and purification	Expensive; preparative throughput is low
GC	Resolves volatile, derivatized toxin classes	Needs derivatization; unsuitable for thermolabile toxins
CCC/CPC	Gentle, solid-phase-free prep for labile toxins	Low resolution; low throughput

## Data Availability

No new data were created or analyzed in this study.
